# Biomimetic Platelet‐Cloaked Nanoparticles for the Delivery of Anti‐Inflammatory Curcumin in the Treatment of Atherosclerosis

**DOI:** 10.1002/adhm.202302074

**Published:** 2024-03-24

**Authors:** Flavia Fontana, Giuseppina Molinaro, Sofia Moroni, Giulia Pallozzi, Mónica P. A. Ferreira, Rubén Pareja Tello, Khalil Elbadri, Giulia Torrieri, Alexandra Correia, Marianna Kemell, Luca Casettari, Christian Celia, Hélder A. Santos

**Affiliations:** ^1^ Drug Research Program Division of Pharmaceutical Chemistry and Technology Faculty of Pharmacy University of Helsinki Helsinki FI‐00014 Finland; ^2^ Department of Biomolecular Sciences School of Pharmacy University of Urbino Carlo Bo Urbino I‐61029 Italy; ^3^ Department of Pharmacy University of Chieti‐Pescara “G. D'Annunzio” Via dei Vestini 13 Chieti I‐66100 Italy; ^4^ Department of Chemistry University of Helsinki Helsinki FI‐00014 Finland; ^5^ Institute of Nanochemistry and Nanobiology School of Environmental and Chemical Engineering Shanghai University Shanghai 200444 P. R. China; ^6^ Laboratory of Drug Targets Histopathology Institute of Cardiology Lithuanian University of Health Sciences Kaunas LT‐44307 Lithuania; ^7^ Department of Biomaterials and Biomedical Technology University Medical Center Groningen, University of Groningen Groningen 9713 AV The Netherlands; ^8^ Present address: MedEngine Oy Eteläranta 14 Helsinki 00130 Finland

**Keywords:** anti‐inflammatory, atherosclerosis, biohybrid, nanoparticles, platelets membrane

## Abstract

Atherosclerosis still represents a major driver of cardiovascular diseases worldwide. Together with accumulation of lipids in the plaque, inflammation is recognized as one of the key players in the formation and development of atherosclerotic plaque. Systemic anti‐inflammatory treatments are successful in reducing the disease burden, but are correlated with severe side effects, underlining the need for targeted formulations. In this work, curcumin is chosen as the anti‐inflammatory payload model and further loaded in lignin‐based nanoparticles (NPs). The NPs are then coated with a tannic acid (TA)− Fe (III) complex and further cloaked with fragments derived from platelet cell membrane, yielding NPs with homogenous size. The two coatings increase the interaction between the NPs and cells, both endothelial and macrophages, in steady state or inflamed status. Furthermore, NPs are cytocompatible toward endothelial, smooth muscle and immune cells, while not inducing immune activation. The anti‐inflammatory efficacy is demonstrated in endothelial cells by real‐time quantitative polymerase chain reaction and ELISA assay where curcumin‐loaded NPs decrease the expression of *Nf‐*κb, TGF‐β1, IL‐6, and IL‐1β in lipopolysaccharide‐inflamed cells. Overall, due to the increase in the cell−NP interactions and the anti‐inflammatory efficacy, these NPs represent potential candidates for the targeted anti‐inflammatory treatment of atherosclerosis.

## Graphical abstract

Biohybrid nanoparticles (NPs) are developed for the delivery of anti‐inflammatory curcumin in atherosclerosis. Lignin NPs are coated with a tannic acid (TA)‐iron (III) complex and cloaked with fragments of platelet membranes. The particles are cytocompatible and decrease the expression of pro‐inflammatory genes in LPS‐induced inflamed endothelial cells, while the cloaking increases the interaction with the target cells.

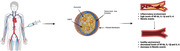

## 1 Introduction

Atherosclerosis‐derived cardiovascular diseases still represent one of the leading causes of morbidity and mortality worldwide, not limited to Western countries or older men, but being increasingly found in younger patients and women.^[^ ^1^, ^2^ ^]^ Together with traditional risk factors, such as high cholesterol levels, inflammation has been recognized as a fundamental participant in the development of the atherosclerotic plaques and in disease progression.^[^ ^3^, ^4^ ^]^ Types of immune cells play a role in the pathogenesis or maintenance of atherosclerosis, from tissue‐resident macrophages to circulating monocytes, to antigen specific CD8 and CD4 T cells.^[^ ^4^ ^]^ Thereby, the research of possible treatments for the disease has been extended from cholesterol‐lowering mechanisms to the immune system. For example, tolerogenic vaccines based on disease‐specific peptides are being evaluated in preclinical studies,^[^ ^5^ ^]^ while clinical studies involving the administration of anti‐IL 1β (CANTOS study) or colchicine have demonstrated the efficacy of anti‐inflammatory therapies in decreasing the number of cardiovascular events.^[^ ^1^ ^]^ However, these clinical studies have also highlighted the risks associated with a systemic, non‐targeted, anti‐inflammatory therapy resulting in moderate and severe adverse events (e.g., infection and sepsis).^[^ ^6^ ^]^

Nanoparticles (NPs) can provide local or targeted delivery of therapeutic molecules to sites of interest, including the atherosclerotic plaque, opening doors to existing treatments with an unfavorable toxicity profile when administered systemically.^[^ ^7^ ^]^ It has been demonstrated both in mice and primates that the successful delivery of a small hydrophobic anti‐inflammatory molecule by high‐density lipoprotein‐reconstituted NPs prevented the activation of the circulating monocytes and macrophages at the plaque level.^[^ ^8^ ^]^ Polymeric NPs have been widely employed for the local delivery of anti‐inflammatory molecules in the intestine, lungs, or to the atherosclerotic plaque, with enhanced efficacy compared to a systemic administration.^[^ ^9^, ^10^, ^11^, ^12^ ^]^ Lignin, a biopolymer derived from the processing of wood pulp, has been investigated as a green alternative to other polymers for the production of high value NPs for drug delivery.^[^ ^13^ ^]^ Our group has explored the use of lignin for the formulation of chemotherapeutics and immunotherapy for the treatment of cancer, and for the transintestinal delivery of insulin in diabetes.^[^ ^14^, ^15^, ^16^ ^]^

The formulation of therapeutic molecules within NPs, albeit displaying advantages and lower toxicity, is not a guarantee for an effective local delivery of the particles only at the target site.^[^ ^17^ ^]^ NPs administered intravenously tend to accumulate within the liver and spleen, or are flagged from the immune system as foreign bodies, speeding their removal from the circulation and reducing the fraction of the therapeutic dose delivered at the diseased site.^[^ ^18^ ^]^ In the last decade, a top‐down approach to prolong the circulation time and the targeting of NPs has been developed by cloaking the particles with fragments of membranes derived from cells.^[^ ^19^ ^]^ The source of the cell membrane chosen for the coating determines the type of targeting obtained: either homotopic targeting (e.g., targeting the source cell, as in the case of cancer cells)^[^ ^20^ ^]^ or general targeting to the site of inflammation (atherosclerotic plaque). Platelets represent an easy access, universal source of membrane while providing increased circulation time, targeting inflamed sites, and interacting with damaged vasculature.^[^ ^19^ ^]^

Curcumin is a polyphenol derivative from turmeric (*Curcuma longa*) with a long use in traditional medicine.^[^ ^21^ ^]^ Curcumin has shown efficacy in the treatment of different cardiovascular diseases through interaction with several targets and cascades at cellular level.^[^ ^22^, ^23^ ^]^ As for atherosclerosis, curcumin plays a role in the cholesterol homeostasis, in the expression of TLR4 receptors, and in the P38‐MAPK, JNK and NF‐κB pathways, amongst others.^[^ ^22^ ^]^ Furthermore, curcumin can actively modulate macrophages within the atherosclerotic lesion from M1 to M2 phenotype.^[^ ^24^ ^]^

**Scheme 1:**
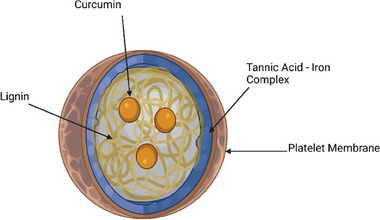
Schematic illustration of Curc@Lignin@TA@PL. Curcumin was encapsulated in lignin NPs. The particles were then coated with a TA−iron complex, followed by cloaking with fragments of platelet membrane. Image created with Biorender.com.

Thereby, the main aim in this work was to load curcumin as an anti‐inflammatory small hydrophobic molecule within lignin‐based NPs aiming to decrease the inflammation in atherosclerosis. Furthermore, the NPs were first coated with a tannic acid (TA)−iron (III) complex with anti‐inflammatory and antifibrotic properties, as well as targeting ability to M1 macrophages within the plaque, followed by cloaking with membrane fragments derived from platelets (**Scheme** **1**).^[^ ^25^, ^26^, ^27^ ^]^ We investigated the cytocompatibility both in human coronary artery endothelial cells HCAEC and in immune cells, followed by the assessment of the interactions between the particles and healthy cells or cells cultured in pro‐inflammatory conditions. Finally, we evaluated the immunological profile of the empty NPs on macrophages and peripheral blood monocytes and the anti‐inflammatory efficacy of the developed nanosystem on HCAEC.

## 2 Results and Discussion

### 2.1 NPs Formulation and Physicochemical Characterization

Lignin NPs were produced by solvent exchange through dialysis against ultra‐pure water, as previously reported, with an optimal concentration of lignin of 15 mg mL^−1^ yielding uniform particles.^[^ ^15^ ^]^ Curcumin was loaded within the particles by dissolving it in tetrahydrofuran (THF) together with lignin before a solvent exchange step. The initial concentration of curcumin in ratio to lignin was investigated as variable for the production of NPs with lower size and polydispersity index (PdI). As shown in Figure S1 (Supporting Information), a high feeding concentration of curcumin (1 mg of curcumin to 10 mg of lignin) produced particles with sizes of 387±61 nm and PdI 0.399±0.059, despite leading to a loading degree (L.D.) of 8.26%. On the contrary, lowering the feeding concentration of curcumin to 1 mg every 15 or 20 mg of polymer yielded particles with size ≈320 nm and PdI of 0.22. All the NPs formulated presented negative ζ‐potential values higher than ‐30 mV (from −33 to ‐36 mV). The ratio curcumin:lignin 1:15 was selected over the ratio 1:20 to guarantee a higher loading degree, together with the use of less polymer, important both in terms of biocompatibility and of final cost of the formulation. The loading degree of curcumin in particles produced with the 1:15 ratio was 6.7%, while it decreased to 3.52% for the 1:20 ratio.

**Figure 1:**
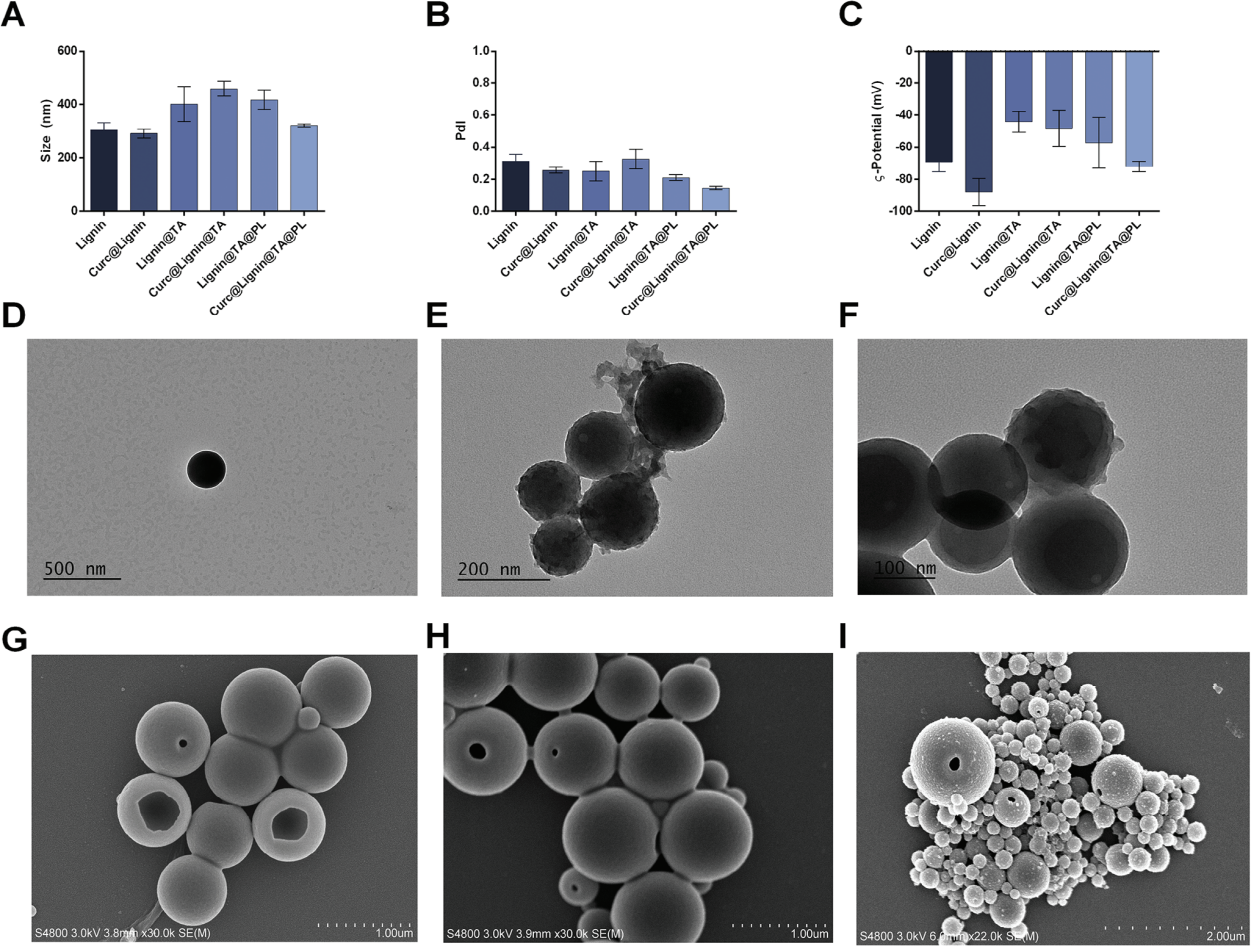
Physicochemical characterization of the formulation. A) Size (nm); B) PdI; and C) ζ‐Potential (mV). The results are presented as mean±s.d. (*n* = 3 independent replicates, each constituted by three technical replicates). D–F) Transmission Electron Microscope images of Lignin, Lignin@TA, and Lignin@TA@PL, respectively. Scale bars: 500 nm in D, 200 nm in E, 100 nm in F. G–I) Scanning Electron Microscope images of Lignin, Curc@Lignin, Curc@Lignin@TA, respectively. Scale bars: 1 µm for G and H, 2 µm for I.

The surface of lignin or curcumin‐loaded lignin NPs (Curc@Lignin) was then further modified to introduce a layer of TA‐iron (III) complex, according to a previously reported methodology.^[^ ^27^ ^]^ The obtained particles (Lignin@TA or Curc@Lignin@TA) were further cloaked with fragments of platelets cell membrane by sonication, yielding Lignin@TA@PL or Curc@Lignin@TA@PL NPs. As shown in **Figure** **1**A, the size of the NPs increased after adding the TA‐iron complex and the platelet membrane layer; in particular, the size of lignin NP increased from 304 to 402 nm after coating with TA‐iron complex, and further to 418 nm after adding the platelet membrane cloak. As for the particles loaded with curcumin, both the initial size and the final size of the system after all the modifications were lower than the empty NPs, while the intermediate step was bigger than the empty NPs (459 vs 402 nm). This phenomenon has been observed in other publications with different polymers and may derive from tighter interactions between the polymeric chains in presence of curcumin.^[^ ^28^ ^]^ The PdI of the final formulations was 0.211 for the empty NPs and 0.145 for the curcumin‐loaded ones (Figure 1B), highlighting the low PdI of the samples. In all the intermediate stages, the PdI value was between 0.25 and 0.32. Finally, all the formulations presented negative surface charge (Figure 1C); the final formulations presented surface charge of ‐57 mV and ‐72 mV for Lignin@TA@PL and Curc@Lignin@TA@PL, respectively. The L.D. of curcumin did not change after the surface modifications; in Curc@Lignin@TA, the L.D. was 5.5%, while after coating with platelet‐derived membranes, the L.D. was 6.3%.

The changes in the morphology of the particles following each step of the surface modification were visible in the transmission electron microscope (TEM) pictures (Figure 1D−F). The smooth surface of lignin NPs (Figure 1D) changed to a rougher one after adding the TA‐iron complex (Figure 1E), while the final cloaking with platelet membranes smoothened the NPs (Figure 1F). The scanning electron microscope (SEM) images (Figure 1G−I) provided insights on the particle formation mechanism, with the partial collapse of one part of the structure to allow for the complete exchange of the organic solvent,^[^ ^29^ ^]^ as well as confirmed the deposition of the TA‐iron complex on the surface of the particle (Figure 1I).

We then quantified the amount of TA and iron deposited on the surface of the particles. First, the indirect quantification of TA in the supernatant, after the complexation and centrifugation to remove the NPs, assessed as UV absorbance at 285 nm, yielded an average of 321 µg of TA per mg of NPs. In order to confirm the results obtained, we processed the supernatants according to the Folin‐Ciocalteu's method.^[^ ^27^ ^]^ The amount of TA quantified in the supernatant was 345 µg of TA per mg of NPs, confirming the results obtained from the measurements based on UV absorbance reported above. Finally, the amount of iron (III) quantified in the supernatant was 53 µg for 1 mg of NPs.

The evaluation of the storability of NPs is fundamental for all the following studies, to determine whether NPs can be produced days before the experiments without any change in their characteristics or if the formulation is not stable over time. As shown in Figure S2A−C (Supporting Information), the final formulation, both empty or loaded with curcumin, was stable for up to 7 days in ultrapure water at +4 °C, maintaining comparable size, PdI and ζ‐potential over time. The intermediate NPs were also stable for up to 7 days in ultrapure water at +4 °C.

**Figure 2:**
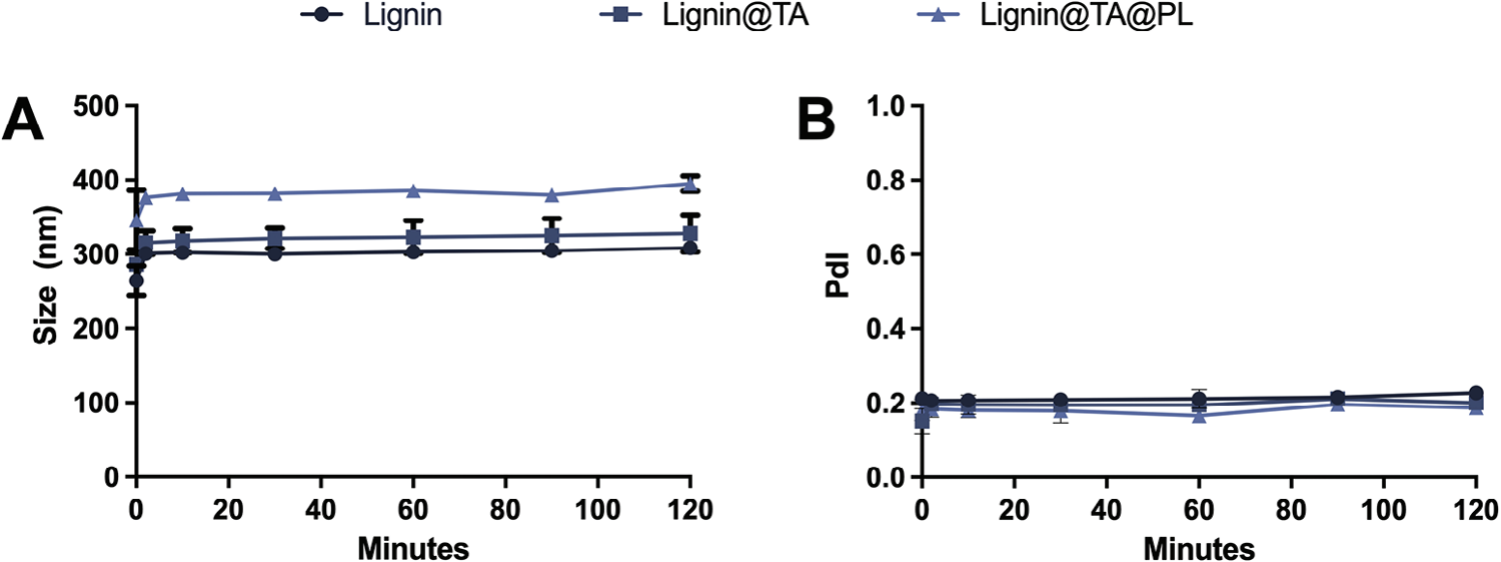
Colloidal stability of Lignin, Lignin@TA, and Lignin@TA@PL in RPMI‐1640 medium with 10% of FBS. A) Size (nm) of the particles over time (0–120 min); and B) PdI of the particles over time (0–120). The results are presented as mean±s.d. (*n* = 3 independent replicates, each constituted by three technical replicates).

The colloidal stability of the formulation is of importance for all the following in vitro studies. In particular, the presence of serum proteins and the formation of a protein corona can determine aggregation of the particles. As shown in **Figure** **2**A,B, Lignin@TA@PL and the intermediate steps were stable for 2 h in RPMI‐1640 medium with 10% of fetal bovine serum (FBS), at +37 °C and under stirring, maintaining size and PdI over time. Lignin NPs were shown to be stable in cell culture media and the coating with the TA‐iron complex has increased the colloidal stability of acetalated dextran‐based NPs.^[^ ^15^, ^27^ ^]^ Finally, the presence of a cloaking with cell membrane can improve the colloidal stability of porous silicon NPs, particularly in presence of proteins.^[^ ^30^ ^]^

Then, we investigated the release of curcumin at pH 7.4 and 5.5, to represent the circulation environment right after administration and the local pH at the site of the atherosclerotic plaque, respectively. As displayed in Figure S3A,B (Supporting Information), at pH 7.4, the payload was released in a burst up to 75% for Curc@Lignin and Curc@Lignin@TA@PL and up to 90% for Curc@Lignin@TA within the first 2 h, with most of the release happening in the first 5–10 min. The burst release from the formulation can be explained both by the pH‐dependent solubility of lignin and by the presence of 1% of Tween‐80 in the buffer solution to maintain the sink conditions throughout the experiment.^[^ ^14^, ^28^ ^]^ The contribution of the polymer pH‐responsiveness was further seen at pH 5.5 in Figure S3C,D (Supporting Information) where the payload was released for ≈50% in a burst fashion within 10 min, followed by a more sustained release up to 24 h. The presence of any of the surface coating did not slow the release at both the pH assessed (5.5 and 7.4).

Overall, curcumin was successfully loaded in lignin NPs, whose surface was then modified to introduce first a TA‐iron complex, followed by fragments of platelet‐derived cell membrane. The NPs were stable at +4 °C for 7 days in ultrapure water and had optimal colloidal stability in RPMI‐1640 medium up to 2 h. Furthermore, curcumin was released in a burst fashion at pH 7.4, while the release, after an initial burst of ≈50%, was sustained up to 24 h at pH 5.5. However, the release profile of the NPs may be poorly suited to the IV administration needed to achieve systemic distribution and interaction with the atherosclerotic plaques.

### 2.2 Cytocompatibility

**Figure 3:**
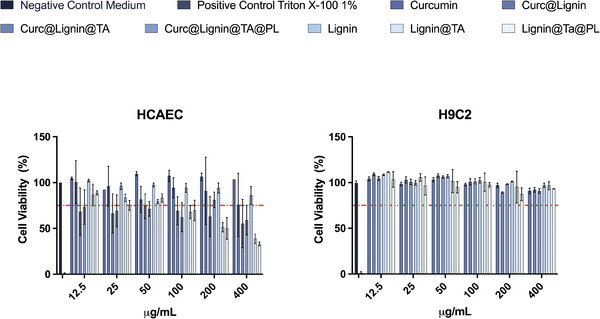
Cytocompatibility in human endothelial cells and in rat myoblasts after 24 h of incubation. A) Cell Viability (%) of HCAEC after 24 h incubation with the final system (Curc@Lignin@TA@PL) and the intermediates. B) Cell Viability (%) of H9C2 after 24 h incubation with the final system (Curc@Lignin@TA@PL) and the intermediate NPs. The results are presented as mean±s.d. (*n* = 3 independent biological replicates, each composed of ≥3 technical replicates). The results were analyzed with two‐way ANOVA, followed by Tukey's post‐test with no statistically significant difference between curcumin‐loaded and empty particles, or the relative intermediates, at all the concentrations assessed. Complete medium and Triton X‐100 (1%) represent the negative and positive control, respectively.

The heterogeneity of the atherosclerotic plaque composition, which includes immune cells, vascular smooth muscle cells, and endothelial cells, widens the range of cell types the NPs will enter into contact with, where the cytocompatibility of the final NPs and all the intermediate steps needs to be assessed to exclude toxicity or undesired confounding factors in the following experiments.^[^ ^3^ ^]^ We selected primary HCAECs as model for the endothelial cells, H9C2 rat myoblasts as models for the smooth muscle cells, KG‐1 as model for macrophages, BDCM and primary, freshly isolated, peripheral blood monocytes as model for monocytes. In HCAEC, Curc@Lignin@TA@PL was safe up to 50 µg mL^−1^ after 24 h of incubation with the cells, as well as all the intermediate NPs (**Figure** **3**A). Furthermore, curcumin, tested in concentration corresponding to the L.D.% of Curc@Lignin@TA@PL, resulted safe for all the concentration assessed. HCAEC cells were previously found to be more sensitive than other endothelial cell types to metal oxide NPs in studies where the highest concentration tested was 200 µg mL^−1^.^[^ ^30^ ^]^ Adding the TA‐iron layer and the platelet membrane coating negatively influenced the compatibility: at concentrations higher than 50 µg mL^−1^, Lignin and Curc@Lignin NPs had optimal cytocompatibility, while for the coated NPs the cytocompatibility decreased, albeit not significantly.

In H9C2 rat myoblasts (Figure 3B), the final system and all the intermediates were cytocompatible up to 400 µg mL^−1^. Further, there was no effect of the coatings on the toxicity of the particles at any concentration assessed. Unlike HCAEC, H9C2 is a permanent cell line, which may explain the lower toxicity recorded compared to the primary HCAEC.

**Figure 4:**
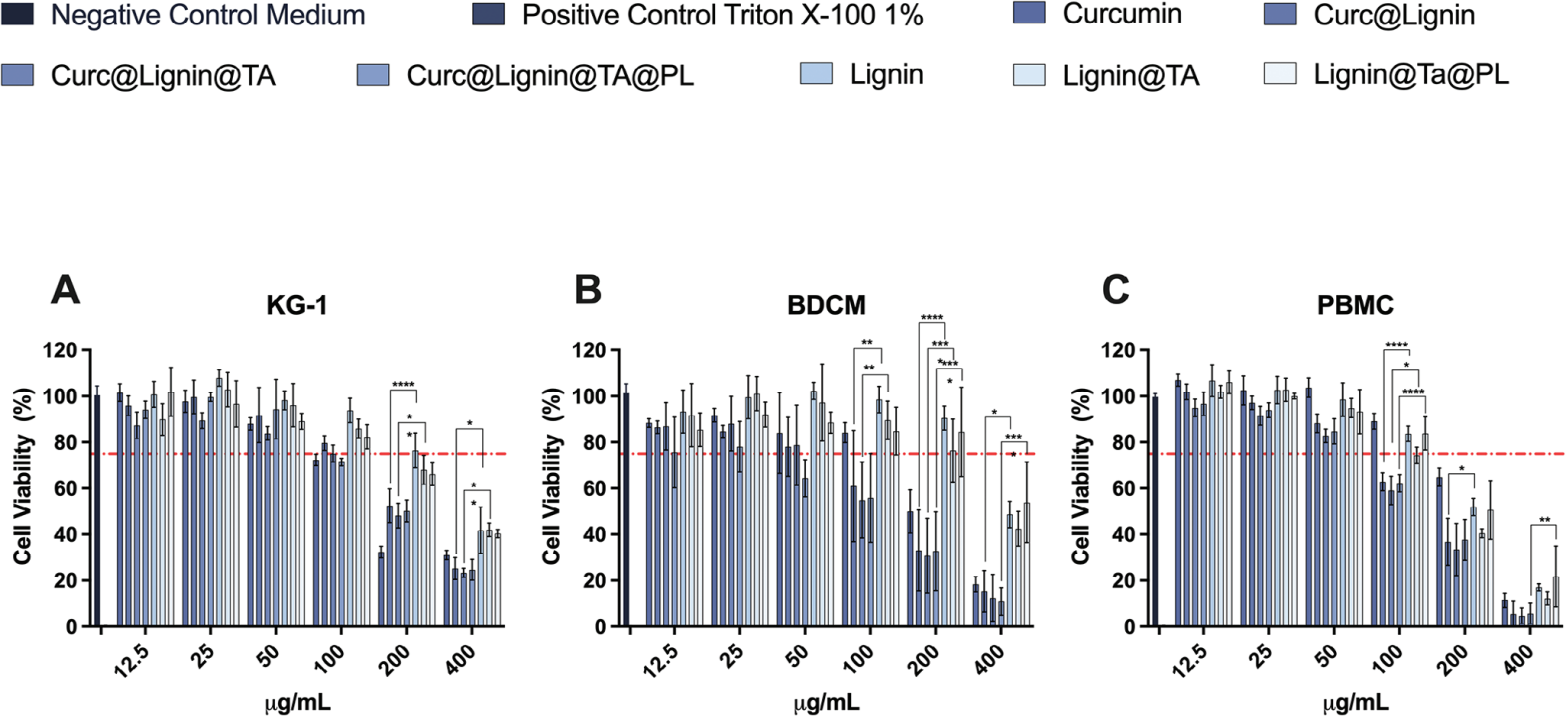
Cytocompatibility in human immune cells after 24 h of incubation. A) Cell viability (%) of KG‐1 after 24 h of incubation with the final system (Curc@Lignin@TA@PL) or the intermediate NPs; B) Cell viability (%) of BDCM after 24 h of incubation with the final system (Curc@Lignin@TA@PL) or the intermediate NPs; and C) Cell viability (%) of PBMC after 24 h incubation with the final system (Curc@Lignin@TA@PL) or the intermediate NPs. Complete medium and Triton X‐100 (1%) represent the negative and positive control, respectively. The results are presented as mean±s.d. (*n* = 3 independent biological replicates, each composed of ≥3 technical replicates). The results were analyzed with two‐way ANOVA, followed by Tukey's post‐test. The cell viability of the loaded final system (Curc@Lignin@TA@PL) was compared to the empty final system (Lignin@TA@PL), the cell viability of the intermediate NPs coated with the TA‐iron complex (Curc@Lignin@TA) was compared to the relative empty NPs control (Lignin@TA), and, finally, the viability of curcumin‐loaded lignin NPs (Curc@Lignin) was compared to empty lignin NPs (Lignin). The levels of significance were set at the probabilities of * p<0.05, ** p<0.01, *** p<0.001, and **** p<0.0001.

In the case of immune cells, the final NPs and the intermediate were safe up to 50 µg mL^−1^ (**Figure** **4**A−C). Contrarily to both endothelial cells and myoblasts, the presence of curcumin in the loaded NPs significantly increased the toxicity of the system in KG‐1, BDCM, and PBMC, compared to empty NPs and intermediates at the same concentration. Curcumin inhibits several pathways within the immune cells, including NF‐κb and STAT.^[^ ^22^ ^]^ For example, in the case of BDCM, their replication is based on the activation of STAT‐1 whose phosphorylation is inhibited by curcumin, leading to cell death.^[^ ^31^, ^32^ ^]^ The proposed mechanism for the curcumin‐induced toxicity in KG‐1 and PBMC is a reduction in the expression of Bcl‐2, which in turn increases the apoptosis in the cells.^[^ ^33^ ^]^ No significant difference was observed in the cell viability between particles in the different stages of surface modification.

Overall, the final system and the intermediate stage of surface modification were cytocompatible up to 50 µg mL^−1^ in all the cells. In the case of endothelial cells (HCAEC), the presence of curcumin was protective toward the particles‐induced toxicity, while in the case of the immune cells, the presence of curcumin further increased the toxicity of the NPs. In the following studies, NPs concentrations found safe were used in the relevant cell line.

### 2.3 NP−Cell Interactions

**Figure 5:**
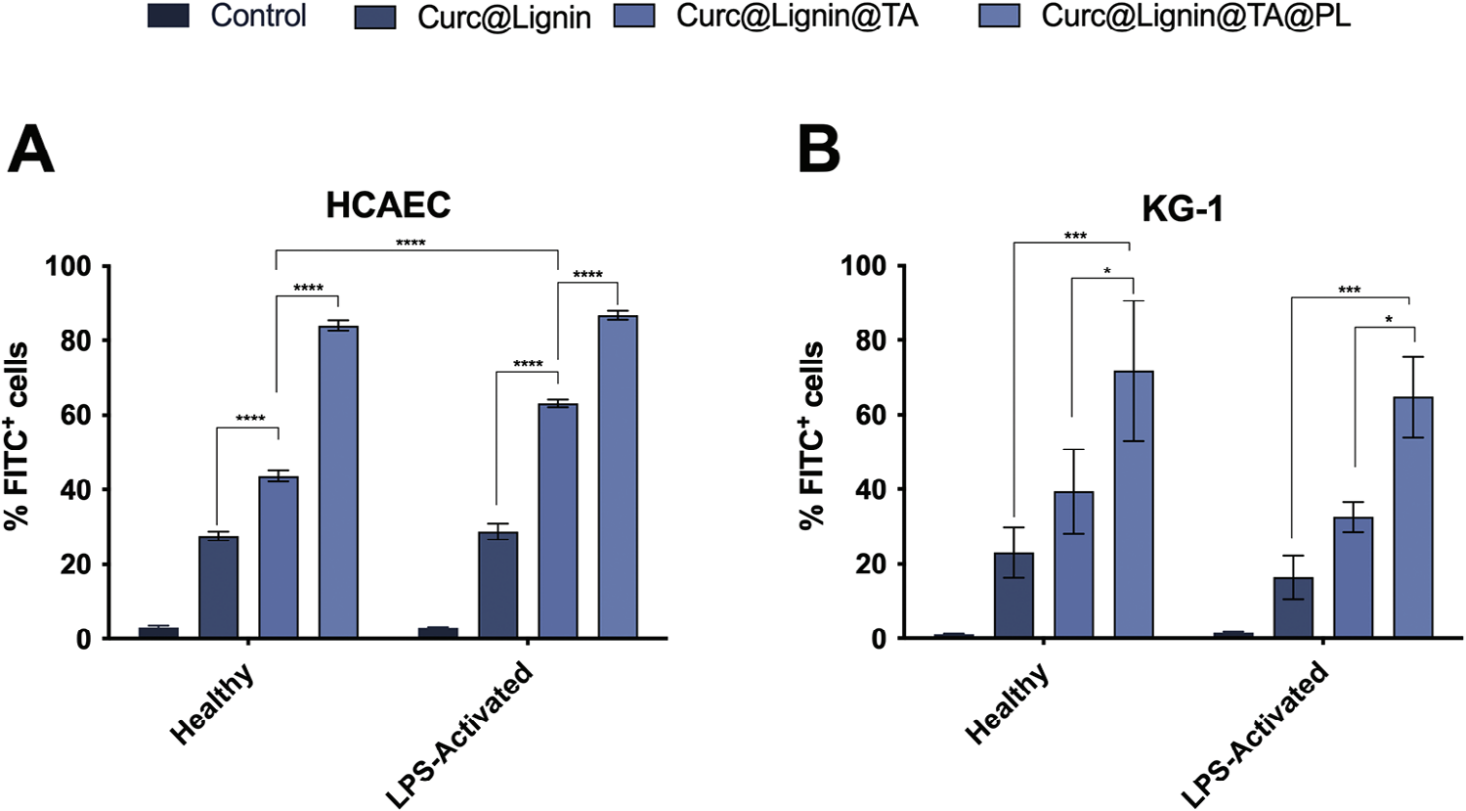
Percentage (%) of FITC^+^ A) HCAEC or B) KG‐1 cells after interaction with Curc@Lignin@TA@PL and intermediate samples for (A) 1 h and (B) 30 min at +37 °C. The NP interaction with the cells, at a concentration of 50 µg mL−1, was evaluated by flow cytometry. The fluorescence of curcumin (loaded within the particles) was used as fluorochrome in the FITC channel. The results are presented as mean±s.d. (*n* = 3 biological replicates, each constituted of 3 technical replicates). The results were analyzed with two‐way ANOVA followed by Tukey's post‐test. The percentage of FITC+ cells in each sample was first compared to the other samples within the same type of cells (healthy vs healthy, e.g., Curc@Lignin vs Curc@Lignin@TA), and then compared with the respective particle in the activated cells (e.g., Curc@Lignin in healthy cells vs Curc@Lignin in activated cells). The levels of probability were set at the probabilities of * p<0.05, *** p<0.001, and **** p<0.0001.

The interaction between negatively charged NPs and cell membrane is generally low when compared with positively charged NPs. The surface modifications introduced on lignin NPs have been proven to increase the interaction between the particles and cells: in the case of the TA‐iron complex, the presence of the complex increased the interaction with primary human tenocytes,^[^ ^28^ ^]^ while the fragments of platelet‐derived membrane increased the interaction with atherosclerotic plaques and damaged vasculature, with cancer cells or pathogens.^[^ ^19^, ^34^ ^]^ The first interaction of interest included the endothelial cells, both in the healthy state and after inflammation, to model whether the particles can interact with the damaged vasculature. As shown in **Figure** **5**A, each surface modification significantly increased the interaction of the NPs, added to the cells at a concentration of 50 µg mL^−1^, with the cells, both in healthy and in LPS‐activated conditions, as assessed quantitatively by flow cytometry. However, the only difference between the interactions in healthy or inflamed conditions was the significant increase of the interaction between TA‐iron‐coated NPs and LPS‐activated cells. The increased interaction in inflamed conditions was present also qualitatively in the confocal microscope images (Figure S4, Supporting Information).

Macrophages, and particularly, inflamed macrophages represent the target cell type of the treatment within the atherosclerotic plaque.[^7^] Thereby, we evaluated the interaction between the particles and KG‐1, a macrophage‐like immortal cell line, both in normal state and after activation with LPS to M1‐like phenotype. As shown in Figure 5B, adding the TA‐iron complex on the surface, and further cloaking the surface with cell membrane significantly increased the fraction of particles interacting with the cells (from ≈20% of Curc@Lignin to 30–40% in Curc@Lignin@TA, and finally to ≥60% in Curc@Lignin@TA@PL). However, no significant differences in the uptake were observed in the interaction between healthy and LPS‐activated cells, suggesting that the interaction is not dependent on the presence of LPS as inflammatory signal. In previous studies,^[^ ^35^ ^]^ the presence of the cloaking with platelet membranes reduced the interaction with macrophages differentiated from THP‐1 cells; however, when the interaction was evaluated with foam cells derived from RAW 264.7 cells, the particles coated with platelet‐derived cell membrane bound the most to the cells.^[^ ^13^ ^]^

Overall, the surface modifications introduced to lignin NPs increased the interaction of the particles with endothelial cells and with macrophages. The presence of the TA‐iron complex was effective in augmenting the number of particles in contact with activated HCAEC cells, while the cloaking with platelet‐derived cell membrane fragments determined the highest interaction with both the cell types, in healthy or LPS‐induced inflammation conditions.

### 2.4 Immunological Profile of the Empty Nanosystem

**Figure 6:**
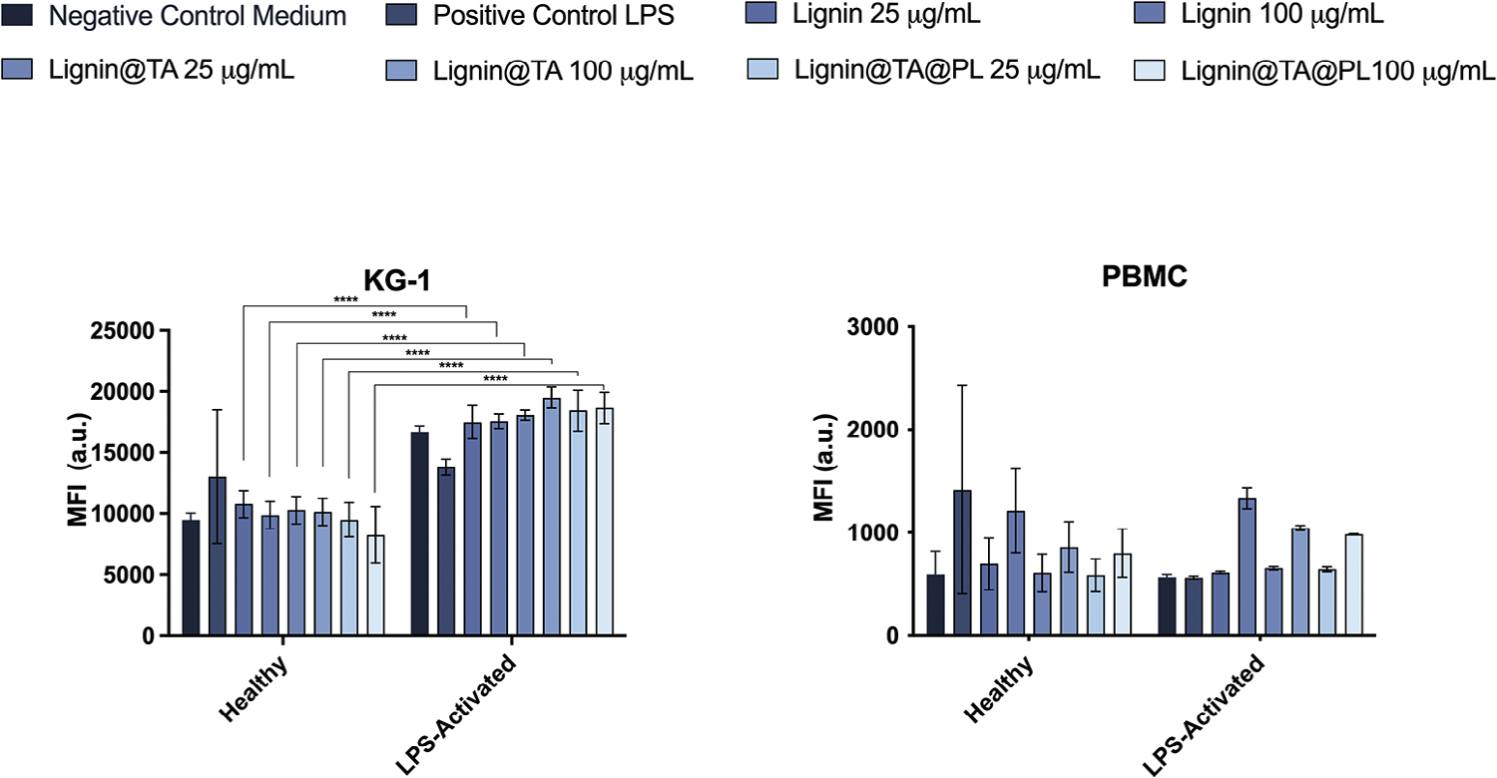
Mean Fluorescence Intensity of APC anti‐human CD86 antibody in A) KG‐1 cells and B) PBMC after 48 h incubation. The cells were incubated with the final empty NP (Lignin@TA@PL) or intermediates for 48 h before being stained for CD86 expression. We run the samples in BD Accuri equipped with a C6 automatic sampler. The results are presented as mean±s.d. (*n* = 3 biological replicates each constituted of three independent technical replicates). The data were analyzed with two‐way ANOVA, followed by Tukey's post‐test comparing all the samples to the control within the same group (healthy or LPS‐activated) or comparing the same sample between the different groups (lignin in healthy cells vs lignin in LPS‐activated cells). The level of significance was set at the probability of ****p<0.0001.

The nanosystem developed in this work is aimed to reduce the inflammation at the level of the atherosclerotic plaque and, specifically, in the activated macrophages within the plaque. Therefore, the NPs and the single components should not elicit a pro‐inflammatory response or further exacerbate the inflammation already ongoing. We evaluated the expression of CD86, a marker of activation, in both KG‐1 macrophages and in PBMC. As shown in **Figure** **6**A, there is no statistically significant increase in the mean fluorescence intensity between the negative control and the samples, at two different particle concentrations (25 and 100 µg mL^−1^), both in healthy and in LPS activated cells. The activation with LPS for 24 h determined an increase in the expression of CD86 across all the samples, with a significant increase when compared with the relevant samples in healthy conditions. Furthermore, the increase of NPs concentration from a low, safe one (25 µg mL^−1^) to the highest safe concentration (100 µg mL^−1^) did not determine an increase in the expression of CD86. As for PBMC, there was no significant difference in the expression of CD86 neither amongst the samples and the controls, nor among healthy and LPS‐activated conditions (Figure 6B).

Overall, the incubation with the particles did not determine an increase in the inflammatory profile of immune cells at both the concentrations tested.

### 2.5 Anti‐Inflammatory Activity in Vascular Endothelium Cells

**Figure 7:**
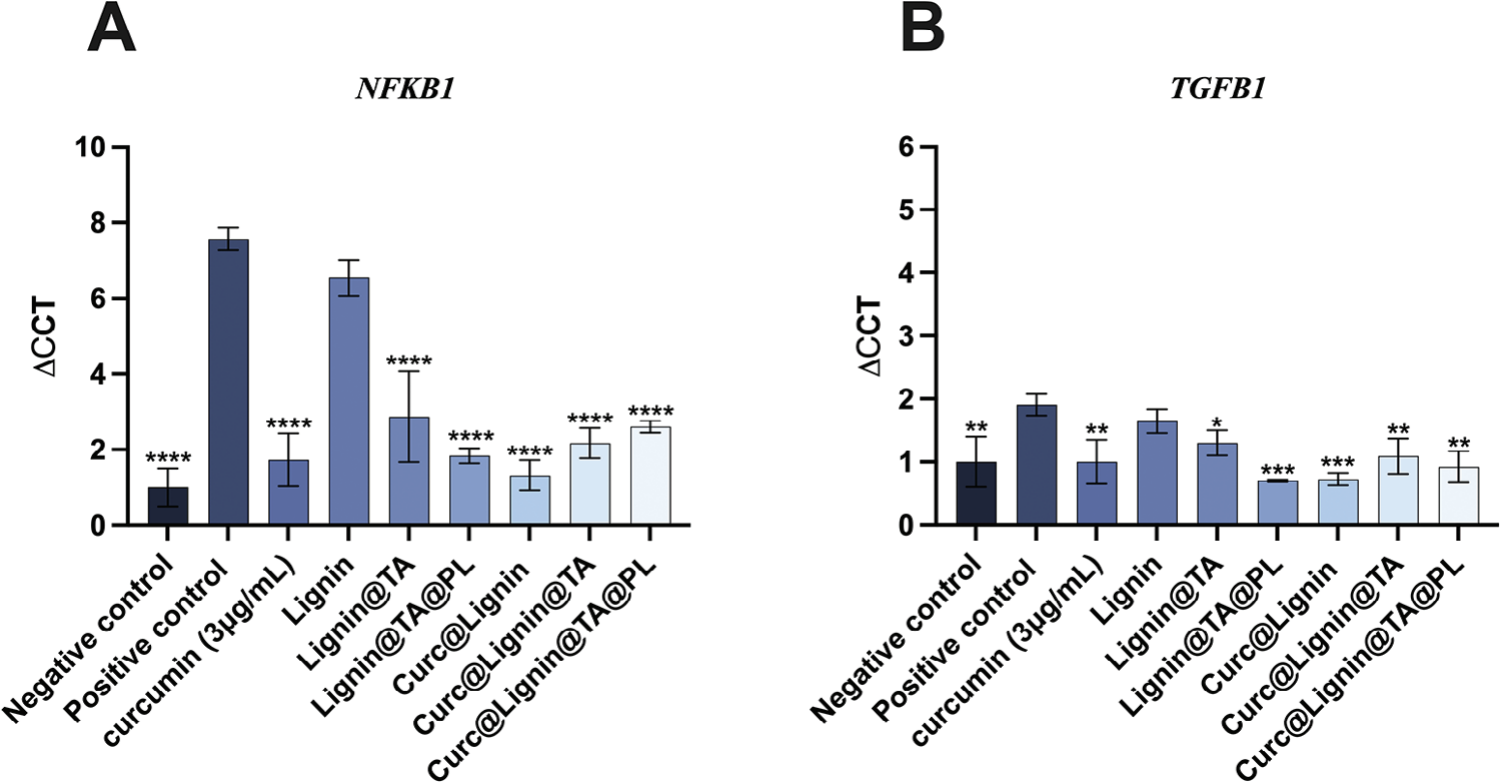
Normalized ΔΔCT of A) NFKB1 and B) TGFB1 in HCAEC cells after inflammation with LPS and incubation with curcumin‐loaded or empty NPs and relative intermediates. The cells were inflamed with LPS (10 ng mL−1) for 24 h, followed by 24 h incubation with the NPs (50 µg mL^−1^). The ΔCCT values were normalized to the housekeeping gene *18s*, and are further normalized by the negative control. The results are presented as mean±s.d. (*n*≥3 independent samples) and were analyzed by paired One way ANOVA versus the positive control LPS. The level of significance was set at the probability of * p<0.05, ** p<0.01, *** p<0.001, and **** p<0.0001.

An important step in the development of a nanoformulation for clinical translation is the initial evaluation of the proposed activity in vitro. Curc@Lignin@TA@PL NPs have been developed to decrease the inflammation at the site of the atherosclerotic plaque. An indirect proof of the anti‐inflammatory activity of the particles was presented by the reduction in cell viability in cells depending on *Nf‐*κb for the replication (Figure 4B). Nevertheless, the evaluation of the efficacy in reducing the expression of *Nf‐*κb and reducing the inflammation is required also in the other cell types forming the plaque, namely endothelial cells. HCAEC cells were inflamed for 24 h with LPS, followed by incubation with curcumin or the NPs for another 24 h. The time point was selected based on the maximum amount of curcumin released (Figure S3A, Supporting Information). Then, we quantified the expression of NFKB1 and TGFB1 by RT‐qPCR. The housekeeping gene *18S* was used as control. As shown in **Figure** **7** A and B, the inflammation of HCAEC was confirmed by the increased levels of expression in the positive control. Furthermore, the incubation of the empty NPs and all the intermediates did not reduce the gene expression of *Nf‐*κb. Contrarily, both curcumin and the curcumin‐loaded NPs significantly reduced the expression of *Nf‐*κb to levels comparable to the negative control (curcumin) or even lower for the NPs, with a progressive decrease in the gene expression after each coating. The decreased expression of *Nf‐*κb in samples incubated with curcumin‐loaded NPs and surface modified is probably due to the enhanced uptake of Curc@Lignin@TA, and even more of Curc@Lignin@TA@PL, by HCAEC in inflamed conditions (Figure 5A). The anti‐inflammatory efficacy of curcumin loaded in NPs has recently been confirmed in primary human tenocytes, with comparable results.^[^ ^28^ ^]^ The coating with TA reduces the expression of TGFB1, suggesting an antifibrotic effect of the formulation, when compared with the positive control treated with LPS, particularly in the samples wrapped with the platelets cell membrane (Figure 7B). A similar trend was observed also in human tenocytes.[^28^]

Overall, curcumin inhibited the gene expression of *NFKB1* and *TGFB1* in HCAEC cells. The encapsulation of the payload within the particles displayed a trend of increased efficacy in decreasing the expression of *NFKB1* and *TGFB1*, which was however not statistically significantly different when compared to curcumin alone.

**Figure 8:**
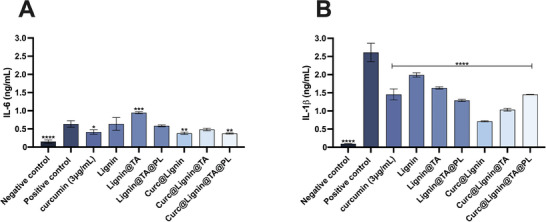
IL‐6 (A) and IL‐1β (B) quantification in the media of HCAEC cells after 24 h incubation with the samples. The cells were pre‐treated for 24 h with LPS (10 ng mL ^−1^) before adding the samples. The results are presented as mean±s.d. (*n*≥3 independent samples) and were analyzed by paired One way ANOVA versus the positive control LPS. The levels of significance were set at the probabilities of * p<0.05, ** p<0.01, *** p<0.001, and **** p<0.0001.

We then evaluated whether the incubation with the particles could influence the secretion of pro‐inflammatory cytokines from LPS‐activated HCAEC. As shown in **Figure** **8**A and B, curcumin can lower the amount of IL‐6 and IL‐1b secreted. Furthermore, the loading of curcumin within the particles (Curc@Lignin@TA@PL) is more effective than curcumin in lowering the secretion, potentially through the enhanced interaction with the cells leading to a higher effect on the *NFKB* signaling pathway.^[^ ^36^ ^]^

As for the secretion of IL‐1b, the multiple activation mechanisms, including inflammasome and lysosomal disruption, play an effect on how the cells react to the final system and each intermediate step. As shown in Figure 8B, the highest inhibition of the secretion is found for Curcumin@Lignin, particles with smaller size and without iron or TA, while the final system (Curc@Lignin@TA@PL) was the least effective amongst all curcumin‐loaded NPs.^[^ ^37^, ^38^, ^39^ ^]^ The exact opposite trend was instead observed for the empty NPs, highlighting a lack of synergistic effect given by the delivery of curcumin with NPs in the final system.

Overall, Curc@Lignin@TA@PL can significantly decrease the secretion of pro‐inflammatory cytokines from LPs‐inflamed HCAEC, with a more pronounced effect on IL‐6 than on IL‐1β.

## 3 Conclusion

In this work we developed biomimetic NPs for the delivery of an anti‐inflammatory compound, curcumin. We loaded curcumin within lignin, a biopolymer derived from wood, and further modified the surface with a TA‐iron complex, followed by cloaking with cell membrane fragments derived from platelets. The obtained NPs showed a homogenous size and negative charge. Furthermore, the particles were stable for 7 days when stored at +4 °C in ultrapure water, while showing excellent stability up to 2 h in cell medium at +37 °C under strong mixing. The payload released in a burst fashion up to 75% within the first 2 h at pH 7.4 and up to 50% at pH 5.5, followed by a more sustained release up to 24 h. The particles were cytocompatible in both endothelial and smooth muscle cells up, while the presence of curcumin determined toxicity at high concentrations in the immune cell models selected. The presence of the surface modifications increased the interaction between the NPs and endothelial cells, both in steady state and in LPS‐inflamed conditions, with similar results recorded for macrophage‐like cells. The developed NPs were not increasing the expression of co‐stimulatory signals in KG‐1 cells. Finally, the efficacy of the NPs in decreasing the inflammation was demonstrated in LPS‐inflamed endothelial cells by RT‐qPCR and ELISA, where the gene expression of NFKB1, TGFB1, IL‐6, and IL‐1b was significantly reduced compared to the positive control.

Overall, the developed NPs formulation can efficiently target endothelial cells and macrophages, while delivering the payload and contributing to lower the expression of inflammatory genes and cytokines. Thereby, this system presents promising properties for a future application in the anti‐inflammatory treatment of atherosclerosis disease. Further research is needed to confirm the mode of action, benefits, and potential drawbacks of the NPs both in vitro and in vivo.

## 4 Experimental Section

### Materials

LigninBoost softwood Kraft was procured from Stora Enso (Helsinki, Finland). Acrodisk Syringe Filter Nylon membrane (13 mm diameter, 0.45 µm pore size) and sterile Acrodisk Syringe Filters with Supor Membrane (13 mm diameter, 0.2 µm pore size) were acquired from Pall Corporation (USA). Tetrahydrofuran (THF, anhydrous, >99.9%, inhibitor free), TA and iron (III) chloride hexahydrate, 4‐(2hydroxyethyl)−1‐piperazineethanesulfonic acid (HEPES), lipopolysaccharide (LPS; from Escherichia coli O111:B4) were purchased from Sigma‐Aldrich (USA). Curcumin was purchased from Santa Cruz Biotechnology (USA). Triton X‐100 was obtained from Merck KGA (DEU). Spectra/Por Dialysis Membrane Standard RC Tubin 12–14 kDa was acquired from Spectrum Laboratories Incorporation (CA). Platelet membranes were isolated from platelets obtained by the Finnish Red Cross from anonymous donors. Ficoll‐Paque was purchased from GE Healthcare (SWE). Cell culture flasks (25 and 75 cm2) were purchased from Corning Incorporation (USA). Fetal Bovine Serum (FBS), Iscove Modified Dulbecco's Medium (IMDM), Recovery Cell Culture Freezing Medium, Trypan Blue, and Penicillin‐Streptomycin‐Glutamine (PEST, 100×) were acquired from Gibco (Life Technologies, USA). Phosphate Buffered Saline (10×), Hank's Balanced Salt Solution (HBSS; 1×), Roswell Park Memorial Institute (RPMI) 1640 Medium, L‐Glutamine (200 mM, 100×), and Non‐Essential Amino Acids (NEAA; 100×) were purchased from Hyclone (USA). Cell Titer‐Glo was purchased from Promega Corporation (USA). APC Mouse Anti‐Human CD86 was obtained from BD Biosciences (USA), while Human BD Fc Block was obtained from Biolegend (USA).

### Lignin and Curc@Lignin NPs Production

Lignin and curcumin‐loaded (Curc@Lignin) NPs were prepared by solvent exchange via dialysis according to a previously reported protocol.^[^ ^15^ ^]^ The aqueous dispersion of NPs was then collected in Eppendorf tubes, centrifuged at 16100 × *g* for 5 min and washed twice to remove residues of THF. The prepared NPs were stored in ultrapure water at the concentration of 1 mg mL^−1^ at +4 °C.

In the optimization phase, different ratios of curcumin:lignin were assessed to identify the best formulation. Namely, the ratios investigated were 1:10, 1:15, 1:20 curcumin:lignin. The formulation with ratio 1:15 displayed the best characteristics and was selected for all further experiments. Curc@Lignin NPs were prepared as reported^[^ ^15^ ^]^ with the following modification. Curcumin (1 mg) was dissolved in the lignin‐THF (15 mg lignin dissolved in 15 mL of THF) solution before adding the solution to the dialysis bag. The curcumin‐lignin‐THF solution was protected from light before, during and after the formation of the particles with foil.

### TA and Iron (III) Complex Formation, Coating of the Particles, and Quantification of the Coating

An aqueous solution of TA (40 mg mL^−1^) and iron (III) (6 mg mL^−1^) was prepared in ultrapure water. Then, 1 mg of lignin or curc@lignin NPs were resuspended in 500 µL of ultrapure water by sonication with a probe sonicator (Vibra‐Cell VCX 500 Ultrasonic Processor, Sonics, USA) for 10 s at an amplitude of 20%. 10 µL of TA solution was added to each sample, followed by vortexing (IKA Vortex 1, Sigma‐Aldrich, USA) for 10 s. Then, 10 µL of iron (III) solution were added to the sample, before vortexing for 20 s. The NPs were then centrifuged for 5 min at 13400  × *g* and resuspended in 1 mL of ultrapure water to obtain lignin@TA or curc@lignin@TA NPs.

The amount of TA coated on the particles was determined indirectly by measuring the absorbance at 285 nm with a Varioskan LUX Multimode Reader (Thermo Fisher Scientific, USA) of the supernatant after the formation of the complex, diluted 1:10 in ultrapure water. A calibration curve (5–160 µg mL−1) with TA was used to determine the amount of TA. Furthermore, the amount of TA coated on the NPs was indirectly quantified also by Folin‐Ciocalteu's method.[^27^] About 100 µL of the supernatant were added to 200 µL of Folin‐Ciocalteu's reagent and mixed with a vortex. Then 800 µL of 0.7 M Na_2_CO_3_ was added and the samples were incubated for 2 h at RT. Then the absorbance of the samples at 765 nm was measured with a Varioskan LUX Multimode Reader. A calibration curve (5–160 µg mL−1) with TA was used to determine the amount of TA. The amount of Fe (III) in the complex was quantified with thiocyanate colorimetry. About 100 µL of supernatant were acidified with 1 M of HCl and were added to 100 µL of 1 M of ammonium thiocyanate solution; the samples were incubated for 15 min at RT and the absorbance at 490 nm was read with a Varioskan LUX Multimode Reader. A calibration curve (5–160 µg mL−1) with iron (III) was used to determine the amount of iron (III) in the complex.

### Isolation of the Platelet Membranes

Platelets were isolated from human blood of healthy anonymous donors, according to the internal blood sampling licenses of the Finnish Red Cross. The experiments were conducted in compliance with laws and with the permission of the anonymous donors. The platelets were isolated as described elsewhere,^[^ ^35^, ^40^ ^]^ aliquoted in Eppendorf tubes (3 × 10^9^ platelets mL^−1^, 1 mL/tube) and stored at −80 °C until further use. Platelet membranes were derived from platelets by repeated cycles of freezing and thawing. The platelet aliquots were collected from −80 °C, thawed at RT, centrifuged at 20800 × *g* for 10 min at +20 °C to avoid platelet activation. The supernatant was discarded, and the platelet pellet was resuspended in 1 mL of ultrapure water. The aliquots were then snap‐frozen in liquid nitrogen. The process was repeated for a total of four freeze‐thaw passages, with the last centrifugation lasting 15 min. The membranes were used immediately for the cloaking of the NPs or stored for up to 72 h at +4 °C.

### Cloaking of NPs with Platelet Membranes

After an initial screening, the optimal concentration of the platelet membranes to obtain the cloaking of lignin@TA or curc@lignin@TA NPs was set at 0.6 × 10^9^ platelets mL^−1^ (data not shown). Lignin@TA or curc@lignin@TA (1 mg mL^−1^) were resuspended by probe sonication for 15 s with 20% amplitude. Then, the platelet membrane suspension (0.6 × 10^9^ platelets mL^−1^) was added to the NPs in a ratio 1:1 in Epppendorf tubes. The samples were bath‐sonicated for 2 min, followed by centrifugation at 5.900 × *g* for 3 min. The cloaked NPs (lignin@TA@PL or curc@lignin@TA@PL) were resuspended in ultrapure water at the final concentration of 1 mg mL^−1^ and stored at +4 °C until further use.

### Physicochemical Characterization of the NPs

The hydrodynamic diameter, polydispersity index (PdI), and zeta (ζ)‐potential of lignin, lignin@TA, and lignin@TA@PL, both empty and loaded with curcumin, were analyzed in a Zetasizer Nano ZS (Malvern Instruments Ltd, UK) by dynamic light scattering (DLS), in disposable polystyrene cuvettes (Sarsted Ag&Co., GER) and in disposable folded capillary cells (DTS1070, Malvern, UK). The samples, at a concentration of 1 mg mL^−1^, were probe sonicated for 5 s at 20% amplitude and diluted 1:100 in ultrapure water. The analysis was run at RT.

The morphology of the particles was imaged with scanning electron microscope (SEM) and transmission electron microscope (TEM). The shape of lignin, curc@lignin and curc@lignin@TA was studied with a SEM. About 10 µL of particle suspension, at the concentration of 1 mg mL−1, were deposited on the surface of silicon wafers and left drying at RT overnight. The samples were then sputter‐coated with gold‐platinum alloy and imaged with a Hitachi S‐4800 (JAP) field emission scanning electron microscope. The morphology of lignin, curc@lignin, curc@lignin@TA, and curcu@lignin@TA@PL was investigated with TEM. About 5 µL of particle suspension, at the concentration of 1 mg mL−1, were deposited on carbon‐coated gold grids (300 mesh, Electron Microscopy Science, USA) and dried overnight at RT. The samples were then imaged with a Jeol JEM‐1400 (Jeol Ltd, JAP) microscope.

### High Performance Liquid Chromatography (HPLC), Loading Degree, Encapsulation Efficiency, and Drug Release Profile

The quantification of the amount of curcumin loaded in the particles, as well as the amount of payload released overtime was performed with RP‐HPLC (Agilent 1100 series, Agilent Technologies, GER) using a 3 µm C‐18 Gemini‐NX column (100 × 4.6 mm, Phenomex, USA), 0.2% phosphoric acid: acetonitrile (45:55%, v/v), flow rate 1 mL min−1, isocratic elution, sampling volume 10 µL, column temperature 25 °C, and detection wavelength 480 nm.

To determine the loading degree (L.D.%, ratio between the mass of the loaded drug and the total mass of drug‐loaded NPs), and the encapsulation efficiency (E.E.%, ratio between the amount of drug encapsulated and the total amount of drug initially added for the encapsulation), 1 mg of curc@lignin, curc@lignin@TA, curc@lignin@TA@PL were added to 2 mL of 99.5% ethanol in Eppendorf tubes and vortexed to dissolve the polymeric matrix of lignin. The samples were then centrifuged at 16100 × *g* for 5 min, the supernatant was collected and diluted 1:10 (v/v) in 99.5% ethanol before being analyzed with HPLC. The in vitro drug release profile at pH 7.4 and 5.5 was evaluated to simulate homeostatic and inflamed conditions, respectively. About 1 mg of NPs were added to the release buffer (phosphate buffer solution (PBS) pH 7.4 + 40 mM of ascorbic acid + 1% w/v Tween80; 2‐(N‐morpholino)ethanesulfonic acid (MES) pH 5.5 + 40 mM of ascorbic acid + 1% w/v Tween80) and stirred at 150 rpm and +37 °C. At predetermined time points, ≈200 µL of samples were taken from each replicate and replaced with a prewarmed fresh buffer. The samples were then analyzed in HPLC.

### Colloidal Stability in Cell Culture Media and Storability in Ultrapure Water

The colloidal stability of lignin, lignin@TA, lignin@TA@PL was studied in cell culture media. The particles were redispersed, at a concentration of 1 mg mL^−1^, in ultrapure water by probe‐sonication for 10 s with amplitude 20%. Then, 180 µL were added to 1 mL of RPMI 1640 medium with 10% of FBS and the particle suspension was stirred at 200 rpm and at 37 °C. At predetermined time points, samples of 180 µL were added to 800 µL of ultrapure water and analyzed for hydrodynamic radius and PdI in DLS. The stability was assessed at 2, 10, 30, 60, 90, 120 min.

The storability of empty and loaded NPs was evaluated over 7 days in ultrapure water. The particles, at a concentration of 200 µg mL^−1^, were resuspended in ultrapure water (2 mL) and kept at +4 °C in static conditions. The storability in terms of hydrodynamic radius, PdI and ζ‐potential was analyzed every 24 h by DLS.

### Peripheral Blood Monocytes (PBMC) Isolation

PBMC was isolated from peripheral blood of healthy anonymous donors, according to the internal blood sampling licenses of the Finnish Red Cross. The experiments were conducted in compliance with laws and with the permission of the anonymous donors. The blood was diluted 1:1 with PBS pH 7.4 at RT. About 15 mL of Ficoll‐Paque reagent was added to a 50 mL Falcon tube. Then, 25 mL of PBS‐diluted blood was carefully added on top of the reagent, to obtain two separate layers. The samples were then centrifuged at 400 *× g* for 40 min, removing the braking at the end of the run. The intermediate layer containing the PBMCs was collected, and it was washed with PBS (pH 7.4), followed by centrifugation at 400 × *g* for 10 min. The washing steps were repeated a total of three times. The PBMC were then placed in a T 75 cm2 flask with 10% of FBS RPMI‐1640 until further use.

### Cell Viability Studies

The cell viability of rat embryonic myoblasts (H9C2), HCAEC, human macrophages (KG‐1), human B cells with dendritic cell morphology (BDCM), and human PBMC was evaluated using a luminescence assay (Cell Titer Glo, Promega, USA). Complete medium (for H9C2, Dulbecco's Modified Eagle's Medium (DMEM) supplemented with 10% of FBS and 1% of PEST; for HCAEC, MesoEndo Cell Growth Medium (MeCG) supplemented with 10% of FBS and 5 ng mL−1 of VEGF; for KG‐1, IMDM supplemented with 10% of FBS and 1% of PEST; for BDCM and PBMC, RPMI 1640 supplemented with 10% of FBS and 1% of PEST) and Triton X‐100 (1%) constituted the negative and positive control, respectively. The adherent cells (H9C2 and HCAEC) were seeded at a density of ≈2 × 10^5^ cells mL^−1^, 100 µL per well, in 96‐well plates (Corning, USA) and left attaching overnight. The medium was removed and the NPs suspensions were added at different concentrations (12.5–400 µg mL^−1^) in complete medium and the plates were incubated for 24 h at 37 °C and 5% CO_2_. After 24 h of incubation, the plates were equilibrated at RT. Then the medium was removed from each well and the wells were washed twice with 100 µL of HBSS−HEPES (pH 7.4). About 100 µL of a 1:1 solution of Cell Titer Glo reagent and HBSS−HEPES was then added to each well. The plates were placed on a shaker for 2 min, followed by 15 min of incubation at RT. The luminescence was then read with a Varioskan Lux Multimode Reader (Thermo Fisher Scientific, USA). As for the cells in suspension (KG‐1, BDCM, PBMC), ≈4 × 10^5^ cells mL^−1^, 50 µL per well, were seeded in 96‐well plates (Corning, USA). Then 50 µL of relevant samples (25−800 µg mL^−1^) were added to each well and the plates were incubated for 24 h at 37 °C and 5% CO_2_. After 24 h of incubation, the plates were equilibrated at RT. About 100 µL of Cell Titer Glo reagent was then added to each well. The plates were placed on a shaker for 2 min, followed by 15 min of incubation at RT. Finally, the luminescence was read with a Varioskan Lux Multimode Reader (Thermo Fisher Scientific, USA).

### Cell Interaction Studies

The interactions between curc@lignin, curc@lignin@TA, curc@lignin@TA@PL were evaluated quantitatively in HCAEC and KG‐1 cells. HCAEC cells were seeded at a density of 3 × 10^5^ cells mL^−1^, 1 mL per well, in 6‐well plates (Corning, USA) and left attaching overnight. The following day, cells were inflamed with LPS (10 ng mL^−1^ in PBS pH 7.4) or left untreated (PBS, pH 7.4). After 24 h, the NPs (50 µg mL^−1^) were added to the relevant wells and they were incubated with the cells for 1 h at 37 °C. Then, the cells were washed twice with HBSS to remove the excess NPs. The cells were then detached from the wells with 800 µL of Trypsin for 2 min, followed by inactivation with 800 µL of trypsin inhibitor and centrifugation at 220 × *g* for 5 min. The cells were then washed twice with 500 µL of HBSS, resuspended in further 500 µL of HBSS, and analyzed with LSR‐II Flow Cytometer (BD Biosciences, USA) in the FITC channel (488 nm laser excitation wavelength). As for KG‐1, the cells were seeded at a density of 3 × 10^5^ cells mL^−1^, 1 mL per well, in 12‐well plates (Corning, USA) and inflamed with LPS (10 ng mL^−1^ in PBS pH 7.4) or left untreated (PBS pH 7.4). After 24 h, the NPs (25 µg mL^−1^) were added to the relevant wells and they were incubated with the cells for 30 min at 37 °C. The cells were then transferred to FACS tubes, centrifuged at 300 × *g* for 5 min, washed twice with 500 µL of PBS. Finally, 500 µL of PBS were added to each tube. The samples were analyzed with LSR‐II Flow Cytometer (BD Biosciences, USA) in the FITC channel (488 nm laser excitation wavelength). The FCS files were then analyzed with Flowjo v.10 (BD Biosciences, USA).

The interaction of the NPs with HCAEC cells was evaluated also qualitatively by confocal microscopy. About 3 × 10^5^ cells mL^−1^, 200 µL per well, were seeded in 8‐well chambers (LabTekSlides, Thermo Fisher, USA) and allowed to attach overnight. The cells were then treated with LPS (10 ng mL^−1^ in PBS, pH 7.4) or sterile PBS (pH 7.4). After 24 h, the cells were treated with the NPs (50 µg mL^−1^), followed by 1 h incubation at 37 °C. Then the cells were washed once with HBSS to remove the excess NPs. Cell Mask Deep Red (5 µg mL^−1^, Life Technologies, USA) was used to stain the cell membranes for 3 min at 37 °C. The cells were washed once with HBSS and fixed with 4% paraformaldehyde (PFA) for 15 min at 37 °C. The excess of PFA was removed with two consecutive washings with HBSS. The cell nuclei were stained with 4′,6‐diamidino‐2‐phenylindole (DAPI, 2.8 µg mL^−1^, blue, Vector Laboratories, USA) for 5 min at 37 °C. Then the chambers were washed 4 times with HBSS to remove the excess DAPI and the samples were stored at +4 °C until imaging. A Leica TCS SP5II HCS A confocal inverted microscope (Leica Microsystems, Germany), equipped with a 405 Diode laser, argon laser (488 nm) and helium‐neon laser (HeNE 633) and an HCX PL APO 63X/1.2 W Corr/0.17 CDS (water immersion) objective was used for the imaging. The images collected were processed using LAS AF (2.6.0 build 7266) software.

### Immunological Profile

The effect of the interaction between NPs and cells on the expression of co‐stimulatory signals (CD86) was investigated in healthy and LPS‐activated KG‐1 and PBMC cells. Cells were seeded at a concentration of 4 × 10^5^ cell mL^−1^, 0.7 mL per well in 12‐well plates (Corning, USA). LPS‐activated cells were treated with LPS (10 ng mL^−1^, in PBS pH 7.4), while healthy cells were incubated with PBS (pH 7.4) for 24 h. Then, the samples were added to the relevant wells and the plates were incubated for further 48 h. The cells were then centrifuged to remove the NPs and transferred to 96‐well V‐bottom plates (Corning, USA). About 2 µL of Human FC Block solution were added to each well, together with 28 µL of PBS. The cells were incubated at RT for 10 min. Then, 7 µL of mouse anti‐human CD86 antibodies were added to each well, together with 93 µL of PBS. The cells were incubated on ice for 20 min. Finally, the samples were centrifuged and washed twice with 200 µL of PBS per well. The cells were then resuspended in 200 µL of PBS and analyzed with flow cytometer (BD Accuri with C6 automated sampler, BD Biosciences, USA) in the APC (640 nm laser excitation wavelength) channel. The FCS files were then analyzed with FlowJo v.10 (BD Biosciences, USA).

### RT‐qPCR and Quantification of IL‐1β and IL‐6 on HCAEC Cells

The anti‐inflammatory effect of curcumin on HCAEC cells was assessed by RT‐q PCR. Briefly, HCAEC cells were seeded in 12‐well plates (Corning, USA) at a density of 6 × 10^4^ cells/well and left attaching overnight. The following day, cells were treated with LPS (10 ng mL^−1^ in HBSS, pH 7.4) or treated with an equal amount of HBSS (pH 7.4) and further incubated for 24 h. Then the samples were added to the cells and incubated 24 h. The RNA isolation was performed using TRIzol (Invitrogen, USA) and the Phase Lock Gel system (5PRIME, lock Gel heavy, QuantaBio, USA), according to the manufacturer's instructions. The cDNA was synthesized using the First‐strand cDNA Synthesis Kit (Transcriptor First strand cDNA synthesis kit, Roche, Germany), before analyzing the RNA with Taqman chemistry in a LightCycler 480 qPCR machine (GE Healthcare Lifescience). The probes used in the assay were acquired from Thermo Fisher Scientific: *18s* (4333760T), Nuclear Factor‐κb (*Nf‐*κb, Hs 00765730‐m1) and Transforming Growth Factor‐b (*Tgfb1*, Hs00998133_m1). The ΔΔCT of each sample was quantified and they were normalized to the housekeeping gene *18S*.

The supernatant of HCAEC samples, was used to quantify the production of IL‐6 and IL‐1β using commercially available ELISA kit: IL‐6 human Elisa kit (**EH2IL6,** Thermo Fisher Scientific, USA) and IL‐1 beta human Elisa kit (**BMS224‐2,** Thermo Fisher Scientific, USA), following the protocol provided by supplier.

### Statistical Analysis

The data were plotted and the statistical analysis was performed in GraphPad Prism 6 (GraphPad Software, Inc., La Jolla, CA, USA). A detailed description of the statistical methods used to analyze the data is reported in each figure legend. In general, ordinary two‐way ANOVA followed by a Tukey's post‐test, was used for the analyses of the different data. For the RT‐q PCR, the data were analyzed with paired T‐test.

## Conflict of Interest

The authors declare no conflict of interest.

## Supporting information

Supporting Information

## Data Availability

The data that support the findings of this study are available from the corresponding author upon reasonable request.
