# Supplementary material for: Biomimetic Platelet‐Cloaked Nanoparticles for the Delivery of Anti‐Inflammatory Curcumin in the Treatment of Atherosclerosis
Source: Adv Healthc Mater. 2024 Mar 24;13(15):2302074. doi: 10.1002/adhm.202302074 (PMC11468963; doi:10.1002/adhm.202302074)
Supplement: Supplementary file 1 — Supporting Information [file ADHM-13-2302074-s001.pdf]

ADVANCED HEALTHCARE MATERIALS

Supporting Information

for *Adv. Healthcare Mater.*, DOI 10.1002/adhm.202302074

Biomimetic Platelet-Cloaked Nanoparticles for the Delivery of Anti-Inflammatory Curcumin in the Treatment of Atherosclerosis

Flavia Fontana, Giuseppina Molinaro, Sofia Moroni, Giulia Pallozzi, Mónica P. A. Ferreira, Rubén Pareja Tello, Khalil Elbadri, Giulia Torrieri, Alexandra Correia, Marianna Kemell, Luca Casettari, Christian Celia and Hélder A. Santos**

Supporting Information

Biomimetic Platelet-Cloaked Nanoparticles for the Delivery of Anti-inflammatory Curcumin in the Treatment of Atherosclerosis

Flavia Fontana[ι,*], *Giuseppina Molinaro*[ι], *Sofia Moroni* [#], *Giulia Pallozzi* [#], *Mónica P. A. Ferreira*[§], *Rubén Pareja Tello, Khalil Elbadri, Giulia Torrieri, Alexandra Correia, Marianna Kemell, Luca Casettari, Christian Celia, Hélder A. Santos*[*]

[*] F. Fontana, G. Molinaro ,S. Moroni, G. Pallozzi, M. P. A. Ferreira, R.P. Tello , K. Elbadri, G. Torrieri, A. Correia, H. A. Santos
Drug Research Program, Division of Pharmaceutical Chemistry and Technology, Faculty of Pharmacy, University of Helsinki, FI-00014, Helsinki, Finland
E-mail: flavia.fontana@helsinki.fi

S. Moroni, L. Casettari
Department of Biomolecular Sciences, School of Pharmacy, University of Urbino Carlo Bo, I-61029, Urbino, Italy

G. Pallozzi, C. Celia
Department of Pharmacy, University of Chieti-Pescara "G. D'Annunzio", Via dei Vestini 13, I-66100, Chieti, Italy

C. Celia
Institute of Nanochemistry and Nanobiology, School of Environmental and Chemical Engineering, Shanghai University, Shanghai 200444, P. R. China

C. Celia
Laboratory of Drug Targets Histopathology, Institute of Cardiology, Lithuanian University of Health Sciences, A. Mickeviciaus g. 9, LT-44307, Kaunas, Lithuania

M. Kemell
Department of Chemistry, University of Helsinki, FI-00014, Helsinki, Finland

H. A. Santos
Department of Biomedical Engineering, University Medical Center Groningen / University of Groningen, 9713 AV Groningen, The Netherlands.

[*] H. A. Santos
W.J. Kolff Institute for Biomedical Engineering and Materials Science, University Medical Center Groningen / University of Groningen, 9713 AV Groningen, The Netherlands.
E-mail: h.a.santos@umcg.nl

[ι,#] These authors contributed equally to the paper.
[§] Current Address: MedEngine Oy, Eteläranta 14, 00130, Helsinki, Finland

Supplementary Data

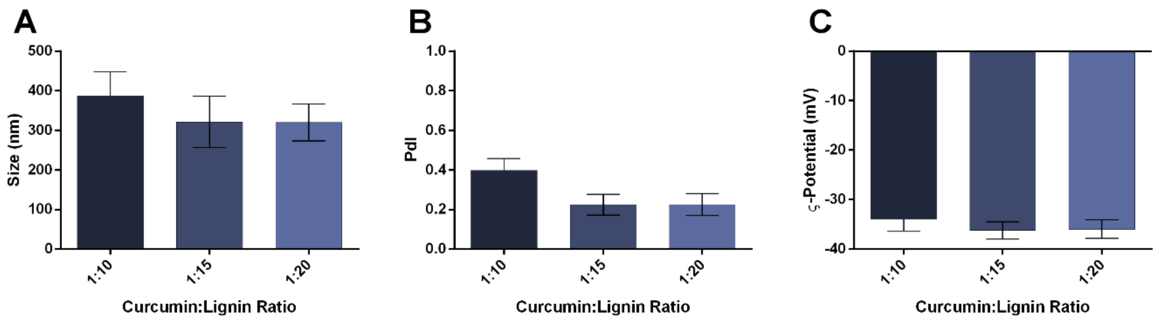

Figure S1. Effect of curcumin feeding concentration on the size and homogeneity of lignin NPs. A) Size (nm), **B)** PdI, and **C)** ζ-potential (mV) of Curc@Lignin NPs prepared from 1:10, 1:15 or 1:20 curcumin:lignin ratio. The results are presented as mean±s.d. (*n*≥3 independent replicates, each constituted by three technical replicates).

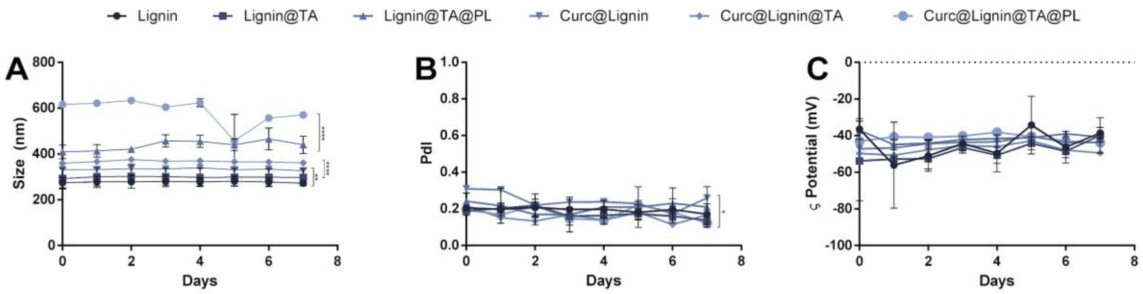

Figure S2. Storability of curcumin-loaded or empty NPs and the relative intermediates at +4°C in ultrapure water. A) Size (nm), **B)** PdI, and **C)** ζ-potential (mV) over time (7 days) of Lignin, Lignin@TA, Lignin@TA@PL, Curc@Lignin, Curc@Lignin@TA, Curc@Lignin@TA@PL in ultrapure water at +4 °C. The results are presented as mean±s.d. (*n*=3 independent replicates, each constituted by three technical replicates). The data were analyzed with one-way ANOVA, followed by Tukey's post-test and the levels of significance were set at probabilities *$p<0.05$, **$p<0.01$, and ****$p<0.0001$.

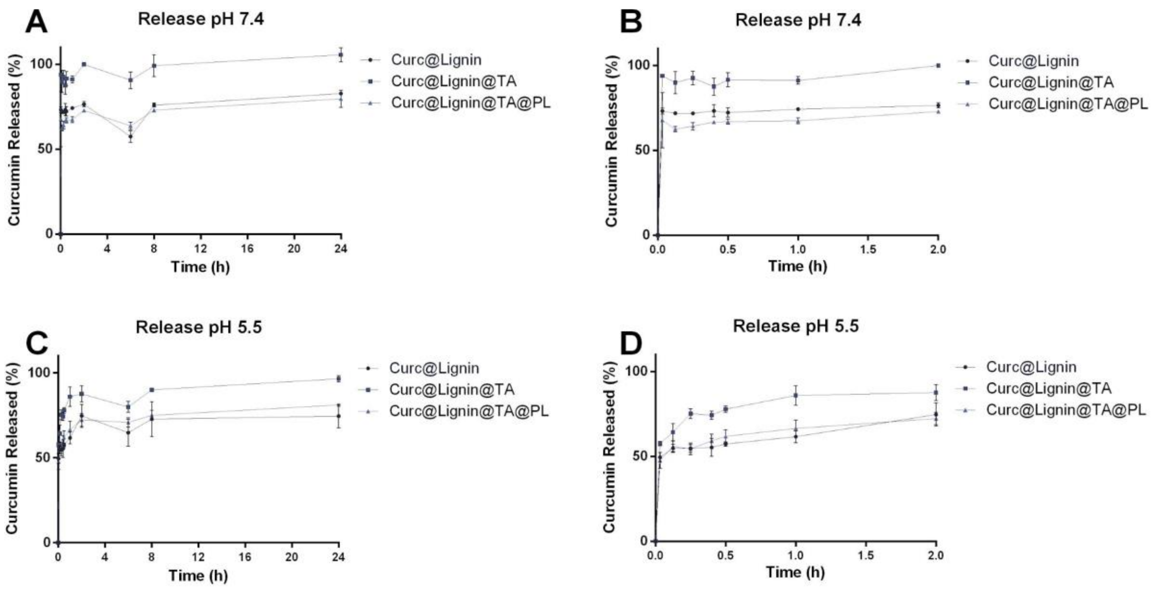

Figure S3. Curcumin release profile at pH 7.4 and 5.5 in sink conditions, at +37°C, under stirring, from the final NPs and the intermediates. A) Release of curcumin over 24 h at pH 7.4; **B)** Release of curcumin over 2 h at pH 7.4; **C)** Release of curcumin over 24 h at pH 5.5; and **D)** Release of curcumin over 2 h at pH 5.5. The release was conducted in sink conditions, at +37 °C, under stirring (300 rpm). The results are presented as mean±s.d. (*n*=3 independent replicates, each constituted by three technical replicates).

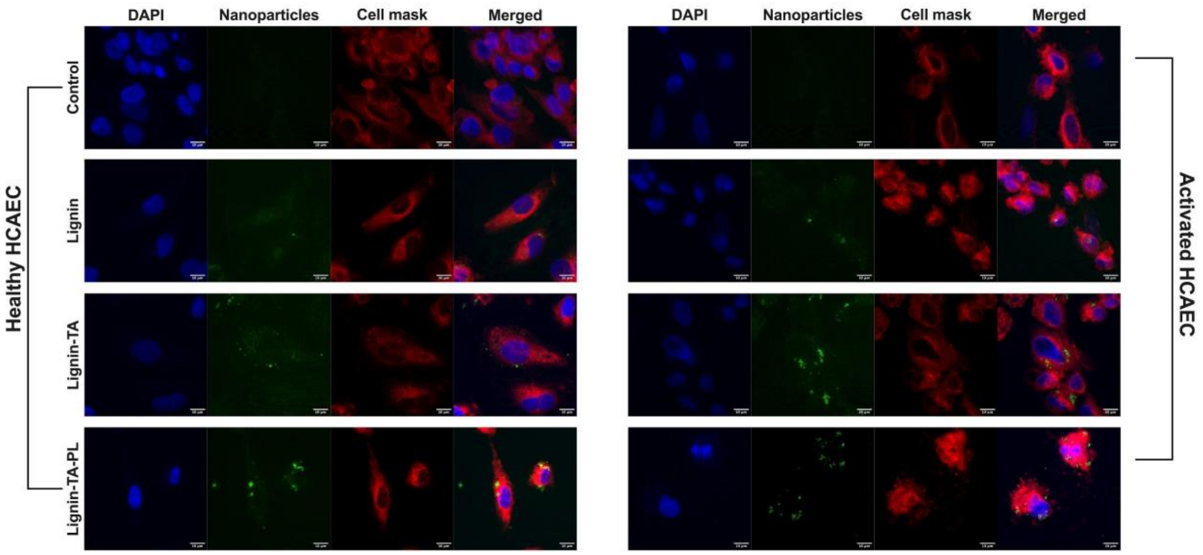

Figure S4. Qualitative uptake of Curc@Lignin@TA NPs and the intermediates in HCAEC cells in steady state or after activation with LPS for 24 h by confocal microscopy.

The uptake was evaluated after incubation with the NPs for 1 h at +37 °C. The curcumin loaded in the NPs was imaged in the FITC channel (Nanoparticles - NPs), while the cell nuclei were stained with DAPI (blue channel) and the cell membranes with Cell Mask Deep Red (red channel). Scale bars 10 μm in all the images.
